# Drag Reduction by Fish-Scale Inspired Transverse Asymmetric Triangular Riblets: Modelling, Preliminary Experimental Analysis and Potential for Fouling Control

**DOI:** 10.3390/biomimetics8030324

**Published:** 2023-07-21

**Authors:** Benjamin W. Hamilton, O. Remus Tutunea-Fatan, Evgueni V. Bordatchev

**Affiliations:** 1 Department of Mechanical and Materials Engineering, Western University, London, ON N6A 6B9, Canada; bhamilt8@uwo.ca; 2 Automotive and Surface Transportation, National Research Council of Canada, London, ON N6G 4X8, Canada

**Keywords:** bioinspired, drag reduction, fouling resistance, functional surface, computational fluid dynamics, precision fabrication, asymmetric triangular riblets

## Abstract

The natural surfaces of many plants and animals provide examples of textures and structures that remain clean despite the presence of environmental fouling contaminants. A biomimetic approach to deciphering the mechanisms used by nature will facilitate the development and application of fouling-resistant surfaces to a range of engineering challenges. This study investigated the mechanism underlying the drag reduction phenomenon that was shown to be responsible for fouling resistance for underwater surfaces. For this purpose, a novel fish-scale-inspired microstructure was shown to exhibit a drag reduction effect similar to that of its natural replica. The primary mechanism through which this occurs is a delayed transition to turbulence. To investigate this mechanism, a Large Eddy simulation was performed at several Reynolds numbers (*Re*). This analysis demonstrated a peak drag reduction performance of 6.7% at *Re* = 1750. The numerical data were then experimentally validated through pressure drop measurements performed by means of a custom-built micro-channel. In this case, a peak drag reduction of 4.8% was obtained at *Re* = 1000. These results suggest a relative agreement between the experimental and numerical data. Taken together, this study advocates that, for the analyzed conditions, drag reduction occurs at low Reynolds numbers. Nonetheless, once flow conditions become more turbulent, the decline in drag reduction performance becomes apparent.

## 1. Introduction

An efficient use of the personal and environmental resources continues to represent the quintessence of life. Due to this, the study of the natural world enables researchers to draw inspiration for innovative solutions to some of the current technical challenges. For instance, biomimicry is the field of science dedicated to the understanding and application of the natural designs for the advancement of human technology. One of the typical examples of biomimicry is constituted by the development of the “flying machines” that have originated from extensive observations of bird flight.

Nature makes use of highly optimized defense mechanisms to mitigate fouling on surfaces. Fouling is generally defined as the unwanted accumulation of environmental contaminants on a surface. Fouling typically leads to the loss of the intended function of the surface on which it occurs. Conversely, fouling resistance is the ability of a surface to prevent the settlement of foulants whereas fouling release implies that certain foulants could be removed after their initial settlement on a surface. Foulants could be biological or inorganic depending on their composition [1]. Biological fouling, or biofouling, consists of both micro- and macro-organisms that preferentially settle and adhere to surfaces that are conducive to their survival [2,3]. On the other hand, inorganic fouling is the result of corrosion, crystallization, suspended particles, oil, and/or ice [1]. Surface fouling is a persistent challenge for many applications including marine transportation, medical systems, and industrial operations like pipelines and water purification [4,5,6,7,8]. The effects of fouling range from significant financial losses—such as in the case of marine transportation—to health risks and even casualties. Therefore, the prevention of undesirable surface buildups constitutes the subject of an active area of research.

The outer surfaces of plants and animals have been observed to employ several mechanisms to mitigate fouling. These include surface energy, chemical secretions, topography, sloughing, and flexion and they can act individually or concurrently to increase the overall efficiency of the fouling resistance [1,9]. For large-scale engineering applications, the most effective fouling-resistant mechanisms appear to involve surface energy and topography, and this is likely a consequence of their passive, low-maintenance nature. The hierarchical microtopography of the lotus leaf constitutes the prototypical example of a fouling-resistant surface in a sense that the tiny structures on the surface of the leaf exhibit a superhydrophobic behavior that enables the water to travel rapidly on the leaf surface. Since water carries away dirt and dust, the end result of this process is a clean leaf surface that essentially facilitates an efficient photosynthesis.

Along the same lines, the microtopography observed on the skin of fast-swimming sharks has received much research attention, largely due to its potential to reduce skin friction drag by nearly 10% [10]. Subsequent studies suggested that drag reduction mechanisms might be associated with fouling resistance [11]. According to the current knowledge, fast-swimming sharks are covered with tiny scales called dermal denticles (toothlike projections), each with several grooves—collectively called riblets—that are parallel to the direction of water flow during shark movement [12]. By contrast, slower moving sharks are covered with similar dermal denticles but the riblets are not present on them such that the shark cannot benefit from their positive drag reduction effects. Early investigations of sharkskin-inspired riblet surfaces discovered that skin friction was concentrated at the low surface area of the riblet tips, with an overall reduction in the riblet valleys. Later studies proposed that optimized riblet geometries were capable of lifting turbulent vortices away from the surface, thus leaving a relatively calm flow inside the riblet valleys [13].

The extent of the drag reduction effect is largely dependent on the riblet spacing relative to the mean vortex diameter. Due to the vortex’s dimensional dependence on fluid parameters and flow conditions, much of the literature reports riblet lengths (period, height, etc.) in non-dimensional wall units denoted with a superscript *+*. For instance, the non-dimensional spacing between riblets, *s^+^*, is defined as:

(1) $$s^{+}=s\frac{u_{\tau }}{\nu },$$

where *s* is the actual spacing between riblets and *v* is the kinematic viscosity. The friction velocity *u_τ_* used in the equation is a commonly used term to denote shear stress in units of velocity. Friction velocity is commonly expressed as $u_{\tau }=\sqrt{\tau_{0}/\rho }$, where *ρ* is the fluid density and $\tau_{0}$ is the shear stress on a flat surface under identical flow conditions. Kim et al. [14] found through direct numerical simulation (DNS) that the mean near-wall vortex has a non-dimensional diameter *d^+^* between 30 and 40. As such, riblets with a spacing less than 30 non-dimensional wall units will prevent the entry of the chaotic vortices into the valleys between adjacent ribs, ultimately resulting in a concentration of skin friction at the riblet tips and an overall reduction in the skin friction drag.

Several investigations of the fouling-resistant properties of sharkskin have concluded that the main functional mechanism is a byproduct of the drag reduction effect. Reduced drag allows the fluid to flow more quickly past the surface, washing away fouling particles that would otherwise settle on, and potentially adhere to, the surface [1,11]. The objective of this research was to determine if the observed connection between drag reduction and fouling resistance is a consistent design trait within nature, thus giving a means of accurately predicting the fouling resistance potential of other bioinspired topographies. Along these lines, the topography of fish scales has also proved to be associated with drag reduction and fouling resistance [5,15]. The underlying mechanism proved to be a delayed transition to turbulence, ultimately leading to water being trapped in the valleys behind the ridges of each scale. Fouling resistance is also present for fish-scale topography. Nonetheless, in this case, the hydrophobic/oleophobic behavior was largely attributed to the thin mucous layer that coats the outer surface of fish scales.

A better understanding of the local hydrodynamic effects that are determined by the microscopic structures present on the surface of various aquatic creatures would enable a superior reverse engineering of nature’s fouling-resistant topographies. To address this, the primary objective of the present work was to provide additional insight on drag reducing mechanisms of bio-inspired fish scales since it is anticipated that they play an important role in the local flow/particle interaction that is ultimately responsible for fouling.

## 2. Computational Model

Numerical simulations have been used extensively to study the drag reduction mechanisms associated with various microtopographies. Three main approaches may be used to solve the Navier–Stokes equations of motion involved in turbulent flow models: Reynolds-Averaged Navier–Stokes, Large Eddy Simulation, and Direct Numerical Simulation. Each of these techniques encompasses a different type of tradeoff between computational time and solution accuracy.

The Reynolds-Averaged Navier–Stokes (RANS) model effectively time averages the equations of motion and introduces turbulence models that are dependent on flow conditions. While the RANS models represent the least computationally expensive approach, investigations of drag-reducing topographies often show weak correlations with experimental data [16,17]. As suggested by its name, the direct numerical simulation (DNS) directly resolves the complete range of spatial and temporal scales of turbulent motion. This requires grid spacings small enough to resolve the smallest turbulent structures—known as the Kolmogorov scales—and small time steps to ensure calculation convergence, therefore requiring significant computing power [18,19]. The large eddy simulation (LES) technique resolves large-scale turbulence while modeling small-scale eddies. Without entering details, it will be briefly reiterated here that as eddy size decreases, the energy contained also decreases. Consequently, the smallest eddies contain the least energy and affect solution accuracy the least, yet they are most computationally difficult to solve. Also, the small-scale eddies are largely isotropic and do not rely on geometry and local flow conditions [20]. LES takes advantage of this by resolving the large-scale eddies and modelling small-scale eddies. This results in a greater solution accuracy than that of the RANS models while obtaining important time reductions with respect to the DNS approach. The LES model has been applied to similar studies on drag-reducing riblets and demonstrates good agreement with experimental data [21,22]. The validation work by Martin et al. with respect to riblets in streamwise flow confirms the applicability of the LES method in modeling of turbulent flows over riblet walls.

### 2.1. Geometry and Characteristics of Transverse Asymmetric Triangular Riblets

**Figure 1:**
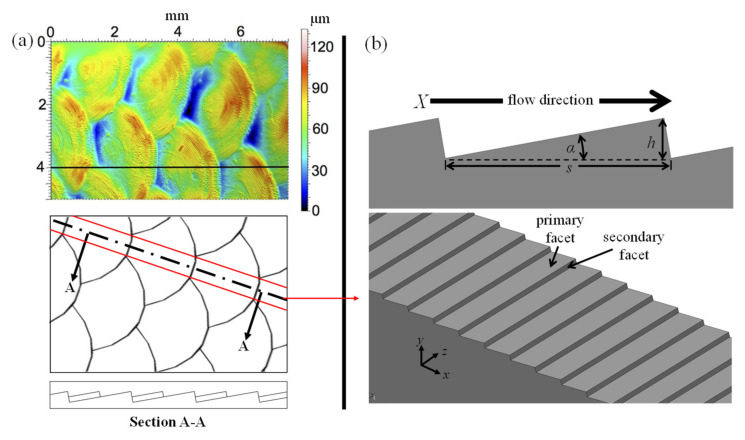
Fish scale geometry: (**a**) natural and (**b**) analyzed.

The geometry considered in this study is inspired by the overlapping pattern of biological fish scales and may be described as a repeating array of asymmetric triangular riblets (ATR) oriented perpendicular to the streamwise direction (i.e., transverse). While the size and shape of fish scales varies considerably between fish species, this study will focus on the topography of the easily identifiable cycloid pattern described by Wainwright et al. [23]. Similar to roofing shingles used in residential housing, each scale of the cycloid pattern overlaps the next in a manner that leaves the semicircular tips of each scale exposed to the flow. Wainwright et al. [23] found that the cycloid scales covering the bluegill sunfish have an average distance between scale tips of 2 mm while the step height ranged between 50 and 100 µm (Figure 1a). It is believed that the dimensional variation of scale size between fish species is largely attributed to their swimming speed and overall body length that in turn depend on the Reynolds number of the aqueous media surrounding the aquatic creature. The ATR geometry used in the present study maintained similar dimensional characteristics as the bluegill sunfish primarily as the means to establish a computational/experimental baseline.

The proposed ATR surface may be considered as a two-dimensional simplification of cycloid fish scales (Figure 1b) in the sense that it is representative of the central region of the scales, denoted as the area framed by the red lines of Figure 1a. By intentionally neglecting the region of scale overlap, in order to investigate the contributing effects of the central region, a more thorough understanding of the hydrodynamic mechanisms of the complex 3D topography is expected to be revealed. The phrase “transverse riblets” or “spanwise riblets” has been implemented in order to reinforce the drag-reducing nature of the proposed topography. Such terminology has been used in the past by [24,25,26,27,28]. The two facets of each asymmetric triangle derive their names from their relative location with respect to the riblet and the corresponding flow direction. More specifically, the primary facet is upwind of the tip, while the secondary facet is downwind of the riblet. The angle between the two facets has been somewhat arbitrarily set to 90° in order to minimize the variables to be considered within this rather preliminary analysis. Therefore, the geometry is fully constrained by the distance between each transverse riblet measured in the flow direction (*s*) and the angle (*α*) between the primary facet and a plane parallel with the surface. The resultant step height between the peak and valleys (*h*) is measured normal to the surface and is a function of the two primary variables:

(2) $$h=\frac{s}{2}\sin(2\alpha )$$

### 2.2. Computational Model

The governing equations of the LES model for continuity and momentum (assuming incompressible flow) are:

(3) $$\frac{\partial \rho }{\partial t}+\frac{\partial (\rho {\bar{u}}_{i})}{\partial x_{i}}=0$$

(4) $$\frac{\partial }{\partial t}(\rho {\bar{u}}_{i})+\frac{\partial }{\partial x_{i}}(\rho {\bar{u}}_{i}{\bar{u}}_{j})=\frac{\partial }{\partial x_{i}}\left(\mu \frac{\partial \sigma_{ij}}{\partial x_{i}}\right)-\frac{\partial \bar{p}}{\partial x_{i}}-\frac{\partial \sigma_{ij}}{\partial x_{i}},$$

where *σ_ij_* is the molecular viscosity stress tensor, and *τ_ij_* is the subgrid-scale stress. The sub-grid stresses are unknown here and must be approximated through a model similar to the one used in RANS models. The present study incorporated the wall adapting local eddy-viscosity (WALE) sub-grid-scale model. This model was developed in response to the shortcomings of other models for wall-bounded flows [20]. The simulation results of the WALE model were shown to have good quantitative agreement with those obtained through DNS and similar experimental studies involving sharkskin-inspired riblets [22].

### 2.3. Domain and Grid Generation

**Figure 2:**
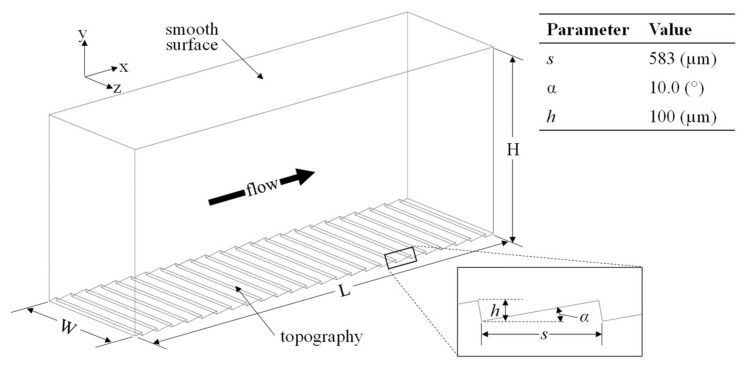
Configuration of computational domain.

Figure 2 depicts the computational domain used in the current study. The domain is delimited by the ATR/riblet-based surface at the bottom along with a flat upper surface that was included for comparative purposes. Jimenez and Moin [29] demonstrated that this type of domain shows a weak correlation between the time-dependent drag on the two surfaces (corr < 0.15). This suggests that the intermittent behavior of instantaneous drag on each surface acts independently of the opposing surface. Both upper and lower boundaries were modeled with a no-slip boundary condition that enables a comparison of the instantaneous drag forces between the two surfaces according to:

(5) $$\Delta D=\frac{D_{r}-D_{s}}{D_{s}}$$

where *s* and *r* subscripts denote the flat and transverse riblet surfaces, respectively. The instantaneous drag force is obtained through integration of the wall shear stress across the surface area. For a surface generating drag forces smaller than those of the flat control/witness surface, Δ*D* will be reported as a negative number. Periodic boundary conditions were applied to the inlet and outlet (*X*-direction) along with the left and right surfaces (*Z*-direction). This particular type of boundary condition has been extensively used in the surveyed literature since it serves as an effective means of simplifying a large domain into a representative “unit” in which turbulent characteristics are accurately captured and modeled.

To ensure computational efficiency, the extent of the physical domain should not be larger than the periodicity of the geometry. However, Jimenez and Moin [29] found that flow conditions drive a lower limit for domain dimensions. This limit is dependent on the relative size of the coherent structures that develop in turbulent flow. As such, if the domain is smaller than this limit, turbulence will be dissipated and the solution will erroneously become laminar. Due to their dependence on flow conditions, domain limits have to be defined in dimensionless wall units to be calculated according to Equation (1). For solution accuracy, the minimal flow unit is 350 wall units in the streamwise (*X*) direction and 100 wall units in the spanwise (*Z*) direction [29]. The domain depicted in Figure 2 is characterized by *L* = 14 mm, *W* = 4 mm, and *H* = 6 mm. The minimal flow unit is satisfied for the five flow conditions considered herein (600 < *Re* < 7840). The Reynolds number for these simulations was based on the channel half height (*δ* = *H*/2) and mean velocity ($\bar{V}$). This Reynolds number corresponds to a friction Reynolds number calculated as:

(6) Reτ=δuv=114

**Figure 3:**
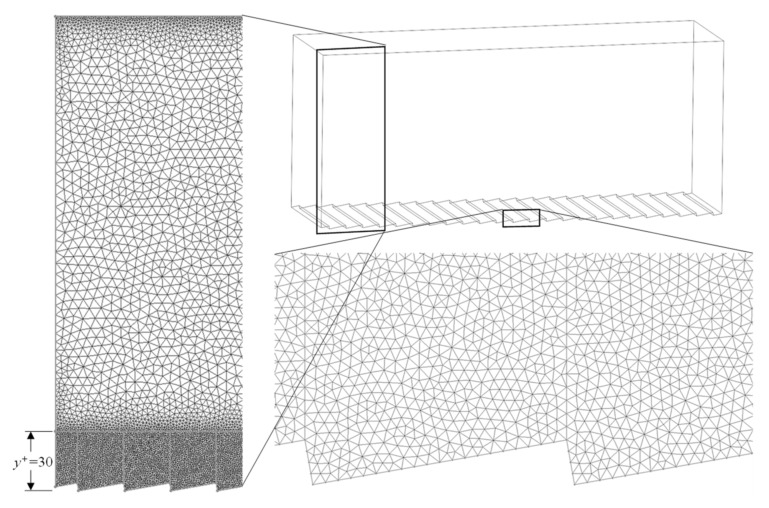
Computational mesh.

The complexity of the ATR surface required the use of non-uniform tetrahedral elements for meshing. The grid spacing was biased toward the two no-slip walls in order to increase the resolution in these areas. The first grid point at both surfaces was *y*^+^ and *x^+^* ≈ 1 and gradually increased toward the center of the domain (*H*/2). As depicted in Figure 3, grid spacing near the bottom surface was kept constant at *y^+^* = 1 up to a height of approximately 30 wall units. The average near-wall streamwise vortex has been shown to have a diameter of approximately 30 wall units with a center of the rotation near *y^+^* = 20 [14]. Therefore, a finer grid resolution in this area is expected to provide a better insight on the effect of ATR structures on these vortices. Nevertheless, the density of the grid was not increased for the upper flat surface because the behavior of near-wall vortices for this type of surface is well-documented in the literature. Due to this, the grid size in the vicinity of the flat surface was chosen to be the same as the transverse riblet surface, and the size of the mesh cells was gradually increased with the distance from the surface. The identical mesh size in the vicinity of the upper and lower boundary enabled direct comparisons of the drag associated with the two types of surfaces (flat vs. structured).

Grid spacing in the spanwise direction was kept to a constant *z^+^* = 1 throughout the domain. Finally, the grid spacing in the streamwise direction is permitted to be as much as *x^+^* = 40 for LES modeling; however, the periodicity of the riblets in the streamwise direction dictated a much smaller spacing (*x^+^* = 1) in order to resolve the geometry. This results in a much higher number of cells in the domain than what is typically reported for simulations with continuous geometry in the streamwise direction (shark-inspired riblets). The number of grid points in the streamwise, wall-normal, and spanwise directions was 468 × 88 × 100, respectively, adding up to a total cell count of 5.6 × 10^6^ cells.

A constant mass flow rate was applied in the streamwise direction. By assuming a parabolic mean velocity profile, the flow rate was estimated by:

(7) $$\dot{m}=\frac{2}{3}\rho A_{c}V_{l}$$

where *ρ* is the fluid density, *A_c_* is the channel cross sectional area, and *V_l_* is the centerline velocity. Following Fluent’s application guideline [20], simulations are initialized by first solving with a RANS model (*k-ε* was used in this study) and then adding turbulent fluctuations to the solution. These become the initial conditions for the LES model whereby the governing equations were integrated forward in time until a statistically steady state is achieved. This was determined by monitoring the instantaneous velocity components at four points in the domain. When the mean velocity and variance were steady, the solution was assumed to be steady. From this point in time, the relevant statistics were gathered across a non-dimensional time interval similar to that used by Martin et al. [21]:

(8) $$T^{+}=T\frac{V_{l}}{\delta }=5000$$

While [21] relied on *T*^+^ = 500, this value was found to be inadequate for the two lowest Reynolds numbers considered in the present study. To address this, a value of 5000 was used to improve the repeatability of the solution.

To ensure the stability of the solution, the time step for each case was chosen to satisfy a Courant value of 1. Approximately 3.0 × 10^5^ timesteps (equivalent to 3000 runtime hours) were required to achieve steady state and obtain the postprocessing data. Since this analysis is computationally prohibitive for most desktop computers, a cluster with 128 cores was used instead. This enabled the reduction of the computation time from months to days.

## 3. Results and Discussion

**Figure 4:**
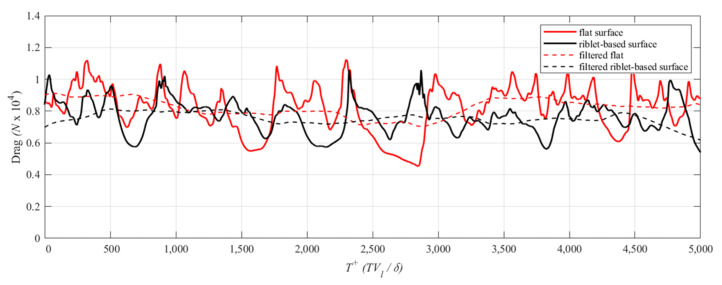
Absolute drag on the top (flat) and functional (transverse riblet-based) surfaces for *Re* = 1750.

The time-dependent evolution of the drag on flat and structured surfaces for the drag-reducing case, *Re* = 1750, is plotted in Figure 4. The time-dependent drag for the flat surface was determined as the area integral of the local shear stress. The periodic nature of the ATR surface in the streamwise direction results in flow separation on the secondary facet. Therefore, the time-dependent drag across the ATR surface is the sum of the shear stress acting tangential to the surface along with the pressure drag acting normal to the surface. The pressure drag is a result of the energy required to move a fluid around an object and depends on the frontal area exposed to the flow as well as its overall shape. The streamlined bodies of many aquatic creatures are an example of shapes that reduce pressure drag. By contrast, friction drag is a result of the shear stress of the fluid acting on a surface parallel to flow direction.

With the exception of brief periods of time, the drag caused by the structured surface is smaller than the drag determined by the flat surface. The intermittent nature of the time history is a result of the small computational domain relative to the length and time scales of the turbulent vortices. Moreover, since the instantaneous drag is an area average, a larger domain would have a dampening effect on the amplitude of oscillations seen in Figure 4; however, the increased number of cells required to discretize such a domain would have a negative impact on computation time. By time averaging across *T*^+^ = 5000, the calculated drag reduction is reported as 6.7%. This amount represents an increase in drag reduction performance compared to prior tests conducted on biological samples of fish scales [30] and similar to the optimal performance of sharkskin-inspired scallop riblets [10].

**Figure 5:**
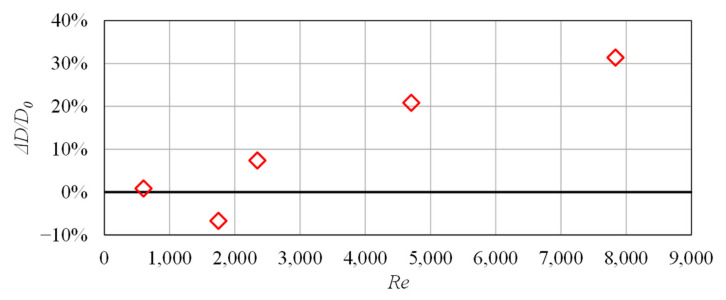
Numerical results outlining the dependence of drag on Reynolds number for the analyzed geometry.

As depicted in Figure 5, a reduction in drag was observed only for the case *Re* = 1750. The remaining cases showed an increase in drag relative to a flat surface. These findings suggest that the mechanism of drag reduction for these structures is a delayed transition to turbulence, similar to that observed for biological fish scales.

### 3.1. Mean Streamwise Velocity Profile

**Figure 6:**
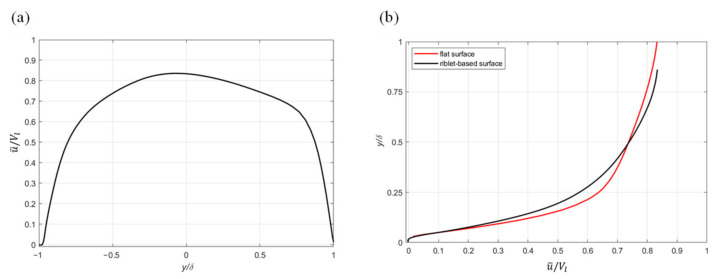
Profile of the normalized mean streamwise velocity: (**a**) across the entire channel height and (**b**) flat vs. structured surface comparison.

The mean streamwise velocity profile for the drag-reducing case, normalized by *V_l_*, is plotted in Figure 6a. The transverse riblet-based and flat surfaces are located at *y/δ* of −1 and +1, respectively. From a qualitative standpoint, the velocity profile in the vicinity of the flat surface (0 < *y/δ* < 1) closely resembles the prototypical turbulent contour, while the velocity profile adjacent to the ATR surface (−1 < *y/δ* < 0) closely resembles the flat, parabolic curve associated with a laminar flow. The difference between the two velocity profiles is consistent with observations of the ability of natural fish scales to delay the onset of the turbulence. Furthermore, the asymmetry of the velocity plot results in a peak velocity shift toward the riblet-based surface. Since a turbulent boundary layer is thicker than its laminar counterpart, the peak velocity shift also suggests an increase in the turbulence near the flat surface compared to the structured surface. In Figure 6b, the origin of the flat surface profile ($\bar{u}/V_{l}=0$) has been assumed to be the riblet peak.

The shear stress on a surface (*τ_w_*) is defined as the product of the fluid viscosity *μ* and the slope of the velocity gradient at the surface ∂u/∂y:

(9) $$\tau_{w}=\mu \frac{\partial u}{\partial y}.$$

The analysis of Figure 6b suggests that the structured surface is characterized by a reduction in the velocity gradient compared to its flat counterpart. Since flow separation results in pressure drag, the overall reduction in drag indicates that the increase in pressure drag in this case is less than the decrease in friction drag.

### 3.2. Turbulent Kinetic Energy Profile

**Figure 7:**
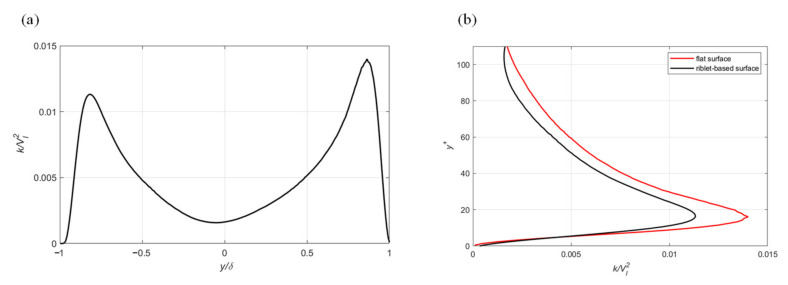
Profile of the normalized turbulent kinetic energy: (**a**) across the entire channel height and (**b**) flat vs. structured surface comparison.

The qualitative observation of the contour of the two velocity profiles from the drag-reducing case suggests that a reduction of the turbulent kinetic energy (TKE) is determined by the riblet-based functional surface. This assumption is further reinforced by the plots of TKE normalized by the mean freestream velocity (Figure 7). Similar to the velocity plots, the transverse riblet and flat surfaces are located at *y/δ* of −1 and +1, respectively. TKE for LES is defined as the sum of its resolved and modeled components [20]:

(10) $$K_{total}=K_{resolved}+K_{sgs}$$

where the subscript *sgs* denotes the modeled portion, indicating it is a function of the *subgrid*-*scale* turbulent viscosity. The resolved portion of TKE is a measure of the energy contained in the chaotic velocity fluctuations of the flow and is defined by:

(11) $$K_{resolved}=\frac{1}{2}({\left(u’\right)}^{2}+{\left(v’\right)}^{2}+{\left(w’\right)}^{2})$$

where the root mean squared velocity fluctuations, in Cartesian coordinates, are denoted as u’, v’, and w’.

A reduction of the turbulent velocity fluctuations near the surface corresponds to a reduction in drag because fluctuations are responsible for momentum transfer from the freestream to the surface. Accordingly, the ATR surface reduces the intensity of turbulence near the surface giving rise to a reduction in the fluid drag. The overlay of the two plots (Figure 7b) illustrates a 19% reduction in peak TKE, and an overall area reduction of 18%. The ratio of resolved turbulent kinetic energy to the total kinetic energy is used to determine the accuracy of the LES results [20]. When 80% or more of the turbulent kinetic energy is resolved, the spatial discretization is considered to be adequate. Since more than 98% of the turbulent kinetic energy was resolved in this study it becomes apparent that mesh cell sizes could be increased in the future in order to reduce the computational time.

**Figure 8:**
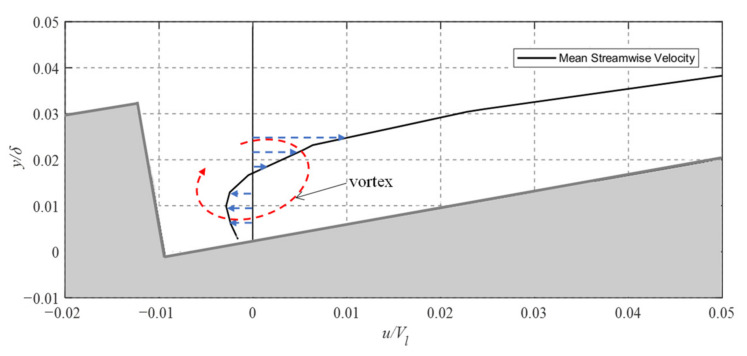
Profile of mean streamwise velocity within riblet valley.

In a recent study of rectangular grooves in transverse flow, the achieved drag reduction was attributed to vortex trapping in the groove valleys [17]. Similar to a moving surface, the counter rotation of the trapped vortices allows the water layer above to slip by more easily than across the stationary facets of each riblet. For the flow conditions and geometry considered here, the center of the trapped vortex is located at approximately 47 µm downstream of the riblet valley and 17 µm above it. Along these lines, Figure 8 depicts a plot of mean streamwise velocity indicating the location of the mean vortex. The vortex is implied by the changing direction of the velocity (the center of the vortex being the location of zero velocity).

### 3.3. Experimental Study

**Figure 9:**
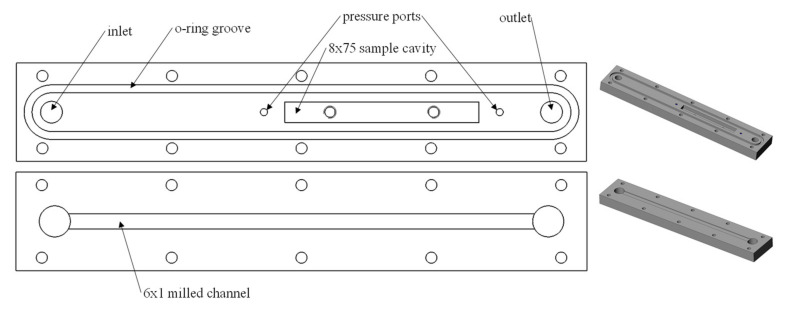
Overview of the microchannel design.

To experimentally validate the numerical results obtained, a closed microchannel was designed and built (Figure 9). This channel enables the measurement of the drop in water pressure caused by the structured sample. Similar setups were used in the past to demonstrate the drag reduction potential of various replicas of naturally structured surfaces [31]. The channel used in this study had a 6 mm width, 1 mm height, and accepts 75 mm long samples to be tested for drag-reduction capabilities. The machined channel was visually examined for defects with an optical microscope and dimensions were measured with calipers. Measured dimensions were used in the relevant calculations. The samples were positioned in such a way to be directly exposed to the flow by essentially forming one of the walls of the channel (typically the upper one). The overall length of the channel was set to be 220 mm to enable a sufficiently long entrance length for flow development purposes. The two halves of the channel were sandwiched together and sealed with a rubber o-ring, forming a closed rectangular channel.

For this experimental setup, the flow was provided by a 12 V centrifugal pump capable of delivering a maximum flow rate of 11.5 L/min. The flow rate was varied with a rotameter (Omega FL-3440ST) such that the velocity ranged from 0 to 3.2 m/s. The rotameter had a respective accuracy and repeatability of ±2% and ± 0.25% of the full-scale. The Reynolds number (*Re*) was calculated with the average velocity in the closed channel. Similarly, the characteristic dimension use to calculate *Re* was defined as the hydraulic diameter, *D_h_*,

(12) $$D_{h}=\frac{4A_{c}}{P},$$

where *A_c_* is the cross-sectional area of the channel, and *P* is the length of its perimeter. The velocity range was chosen such that it encompassed the transition region (Re ≈ 2300) where the ability of the ATR/riblet-based surface to delay turbulence onset would become evident. An eight-liter reservoir was used between the channel outlet and pump in order to mitigate the effects of temperature change on the water viscosity throughout the experiment. As such, the water temperature maintained a consistent 22 °C during the tests. The pressure difference between the two ports shown in Figure 9 was recorded at 1 Hz by means of a handheld General Tools DM8252 differential manometer with a resolution of 10 Pa and an accuracy of 1% of the full-scale reading (13.8 kPa). The flowrate was incrementally decreased at 60 s intervals while recording the pressure drop to facilitate statistical analysis. By comparing the pressure drop caused by the flat sample with that of the ATR surface, drag reduction was observed as a decrease in the pressure required to achieve each incremental flow rate.

**Figure 10:**
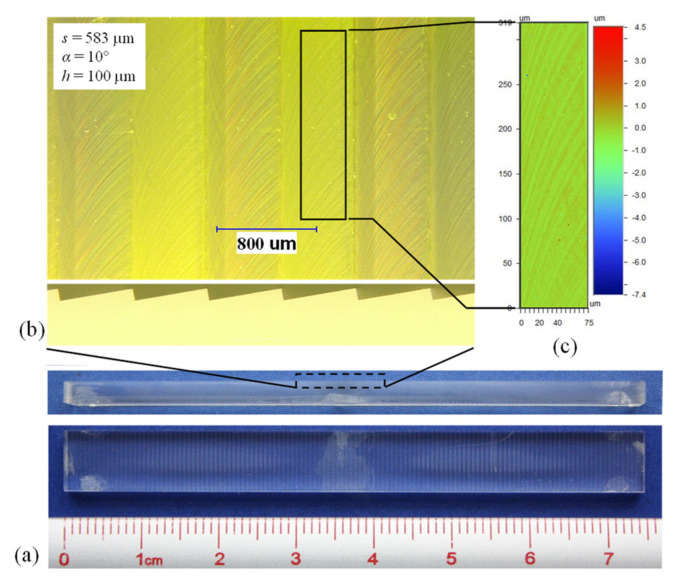
ATR sample: (**a**) overview, (**b**) surface detail, and (**c**) areal surface roughness.

Samples were fabricated from 75 × 8 × 3 mm acrylic stock with a multi-functional Kugler nano5X micromachining system (Kugler GmbH) equipped with a 1 mm diameter monocrystalline diamond endmill. This system is able to achieve optical surface quality (*R_a_* < 10 nm) with an accuracy of +/− 0.5 µm in the plane parallel with the fabricated surface and +/− 0.5 µm normal to the surface. Dimensions of the geometry fabricated for this test were identical to those of the numerical simulation: *s* = 583 µm and *α* = 10°, corresponding to *h* = 100 µm (Figure 10). A reference flat surface was also fabricated for drag comparison purposes.

The form accuracy and surface roughness of the fabricated samples were analyzed with an optical microscope and a white light 3D optical profilometer. The well-defined 90° corners of the riblet peaks and valleys illustrated in the side view of Figure 10b demonstrate the high degree of form accuracy achieved. The mean surface roughness (*S_a_*) was approximately 100 nm. While this is an order of magnitude higher than what is considered optical surface quality, it is deemed to be sufficient for the experimental needs of the current study.

**Figure 11:**
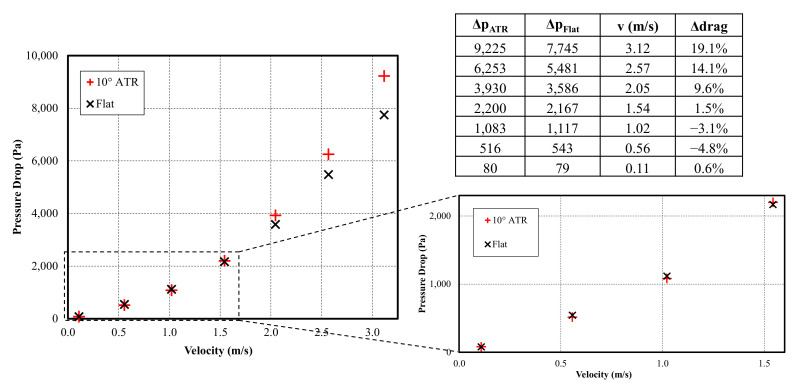
Experimental data suggesting low velocity drag reduction.

Figure 11 outlines the results of the pressure drop experiments for velocities up to 3.12 m/s (equivalent to *Re* = 5500). The propagation of error due to the repeatability of the rotameter and the statistical uncertainty of the pressure measurements (2σ) was calculated for each flow rate. This results in a calculated maximum error of +/− 4.5% of the measured pressure. The reduction of drag was reported for these experiments as a percentage change in the pressure drop with the ATR/riblet-based sample relative to the flat surface. The values reported in the table represent the real change in drag, i.e., a value that accounts for the area fraction. At high flow velocity, it is evident that the ATR-structured functional surface has a higher pressure drop than its flat counterpart. There is, however, a small region at low velocities where drag reduction was present. The peak drag reduction of 4.8% corresponds to a channel velocity of 0.56 m/s and a Reynolds number of 1000. The peak numerical drag reduction of 6.7% was achieved at a similar 0.58 m/s; however, the difference in characteristic dimension of the channel means the optimal performance was achieved at *Re* = 1800.

**Figure 12:**
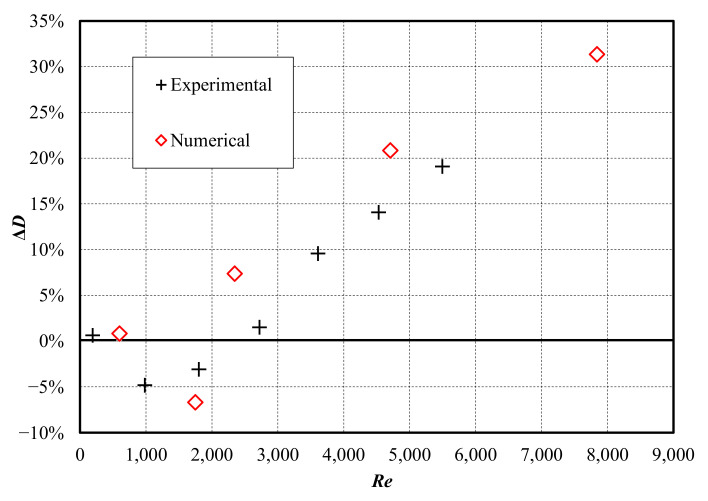
Drag reduction for the analyzed ATR-based functional surface.

The change in drag achieved numerically and experimentally is presented in Figure 12. When expressed in terms of the Reynolds number, the experimental data show a similar trend to the numerical data. Interestingly, the shape of the drag reduction curve is similar to the one obtained in drag reduction studies involving sharkskin-inspired riblets [10]. Peak drag reduction performance was achieved at a lower Reynolds number in the experiment, indicating that the onset of turbulence occurred more quickly than in the numerical simulation. Several factors have probably contributed to this, including surface roughness of the machined channel walls and structured surface and turbulence introduced by the rotating vanes of the pump and pipe network leading into the channel inlet. As drag reduction begins to break down (beyond approximately *Re* = 2000), the experimental results demonstrate a similar slope of increasing change in drag with increasing velocity (*Re*).

The optimal performance for the drag reduction is achieved for a narrow window of Reynolds numbers, suggesting that the effectiveness of these structures is highly dependent on the flow characteristics. In the case of biological fish scales, delaying the onset of turbulence and its associated drag reduces the required energy expenditure for locomotion. While the ATR structures introduced here demonstrate a similar ability to delay turbulence, a corresponding resistance to fouling is also expected since impaction due to the chaotic motion of turbulence contributes to the settlement and adhesion of fouling particles.

## 4. Conclusions and Future Work

Bioinspired asymmetric triangular riblets in transverse flow have been analyzed as a potential fouling-resistant surface topography. The observed correlation between drag reduction and fouling resistance for sharkskin-inspired functional surfaces has been hypothesized as an operational mechanism common to all naturally occurring fouling-resistant surfaces. Accordingly, the drag reduction potential of the ATR-based surfaces constituted the focus of the present study.

The previously reported delayed onset of the turbulence—characteristic to natural fish scales—was also noticed during the numerical simulations performed on the proposed ATR geometry. This phenomenon seems to be a consequence of the water trapped in the vortices developed in the riblet valleys. As these vortices become active, they enable a region of low shear stress for the liquid layers above them. In agreement with these observations, numerical simulations showed a 6.7% drag reduction, whereas the maximum observed drag reduction of 4.8% was achieved experimentally.

The ATR dimensions used throughout this study were chosen to correspond relatively closely to those of natural fish scales. However, a more thorough investigation of the geometry—performed through a parametric study of *s* and *α*—has the potential to reveal optimal dimensions for maximum drag reduction effects. Furthermore, additional numerical investigations of the region where drag reduction effects appear to disappear as *Re* increases are required since they seem to be closely related to the intrinsic nature of the vortices developed in the riblet valleys. Along these lines, it can be speculated here that the drag reduction potential could be extended to a higher *Re* number by increasing the streamwise length of the vortex, i.e., the fraction of surface area it covers on the primary facet. Finally, since the present study verified that the analyzed ATR geometry has drag reduction potential, future studies will focus on the correlation between the drag reduction and fouling resistance that practically represents the end goal of this class of structured surfaces.

## Data Availability

Not applicable.
